# Rab21 Protein Is Degraded by Both the Ubiquitin-Proteasome Pathway and the Autophagy-Lysosome Pathway

**DOI:** 10.3390/ijms23031131

**Published:** 2022-01-20

**Authors:** Pinduo Liu, Anping Wu, Hui Li, Jun Zhang, Junjun Ni, Zhenzhen Quan, Hong Qing

**Affiliations:** Key Laboratory of Molecular Medicine and Biotherapy, School of Life Science, Beijing Institute of Technology, Beijing 100081, China; liupinduo@foxmail.com (P.L.); w18801361945@163.com (A.W.); lixiaohui0918@163.com (H.L.); zhangjun@bit.edu.cn (J.Z.); nijunjun@bit.edu.cn (J.N.)

**Keywords:** Rab21 protein, ubiquitin-proteasome pathway, autophagy-lysosome pathway

## Abstract

Rab21 is a GTPase protein that is functional in intracellular trafficking and involved in the pathologies of many diseases, such as Alzheimer’s disease (AD), glioma, cancer, etc. Our previous work has reported its interaction with the catalytic subunit of gamma-secretase, PS1, and it regulates the activity of PS1 via transferring it from the early endosome to the late endosome/lysosome. However, it is still unknown how Rab21 protein itself is regulated. This work revealed that Rab21 protein, either endogenously or exogenously, can be degraded by the ubiquitin-proteasome pathway and the autophagy-lysosome pathway. It is further observed that the ubiquitinated Rab21 is increased, but the total protein is unchanged in AD model mice. We further observed that overexpression of Rab21 leads to increased expression of a series of genes involved in the autophagy-lysosome pathway. We speculated that even though the ubiquitinated Rab21 is increased due to the impaired proteasome function in the AD model, the autophagy-lysosome pathway functions in parallel to degrade Rab21 to keep its protein level in homeostasis. In conclusion, understanding the characters of Rab21 protein itself help explore its potential as a target for therapeutic strategy in diseases.

## 1. Introduction

Intracellular trafficking is a highly conserved and essential process for vesicular exchanges between organelles and cellular compartments, which contributes to maintaining cellular homeostasis [1,2]. The Rabs (Ras-related proteins in the brain), a family of small GTPase that belongs to the Ras-like GTPase superfamily, are functional as vital regulators of intracellular trafficking [3]. They act as Ras-like small GTPases that their activation can be switched on by GTP-bounding while switched off by GDP-bounding. Rab proteins can regulate vesicular trafficking in different aspects, from membrane budding to vesicle transport and fusion with target organelles [4]. For example, Rab5 can collaborate with various binding partners and control the early endocytic traffic and signaling [5]. Rab7 is found in the late endosome and functional in late endocytic trafficking [6]. Rab2 can be recruited to Golgi and connect Golgi network to autophagy pathway to play a key role in the formation of autophagosome and autolysosome [7]. Rab4, Rab11, and Rab35 involve in the endocytic recycling pathway [8,9,10]. In addition, Rabs are also associated with neuronal functions. It is reported that Rab3 is required for the assembly of the amyloid precursor protein (APP) and kinesin-1C [11] and also interacts with RIM (Rab3-interacting molecule)/Munc13 to approximate synaptic vesicles to the priming machinery [12]. Rab7 is functional in transporting neurotrophins and their receptors, thus acting as a functional marker of a specific pool of axonal retrograde carriers [13]. Rab5 activation is required for the specific internalization of synaptic AMPA receptors, which is crucial for hippocampal long-term depression [14].

Rab21 protein belongs to the GTPase family and is functionally similar to other proteins in this family. It consists of 225 amino acids with a molecular weight of 25 kDa. Rab21 protein can bind to GTP or GDP and present both active and inactive states. Rab21 resides in the early endocytic pathway and is associated with many functions in membrane trafficking, including regulation of endosome morphology and function [15], modulation of cell adhesion, endosomal trafficking of integrins [16], and endosomal sorting of specific clathrin-independent cargo and autophagy [17]. Rab21 is also involved in many diseases. In acute pancreatitis, overexpression of *RAB21* promotes the caerulein-induced MKK3 (mitogen-activated protein kinase kinase 3)-TRAF3 (TNF receptor-associated factor 3) association. It produces pro-inflammatory cytokines, while knockdown or silencing *RAB21* can alleviate the cytotoxicity of caerulein-activated BMDMs to co-cultured pancreatic acinar cells [18], suggesting its role in the pro-inflammation response. It is also reported that *RAB21* acts as an oncogene in glioma cells that downregulation or silencing *RAB21* can suppress the cell growth and induce cell apoptosis in glioma cell lines [19]. Recently, Rab21 has been identified as a novel interactor of PS1, one of the key secretases for hydrolyzing APP protein to produce beta-amyloid (Aβ) and regulate the activity of PS1 by transferring it from early endosome to late endosome/lysosome, suggesting its crucial role in the neuropathogenesis of AD [20]. However, how Rab21 itself is modulated is still unknown. In this study, we examined the proteolytic pathways involved in the degradation of Rab21. Revealing the proteolytic pathways of Rab21 will help us better understand and further explore the functions of Rab21.

## 2. Materials and Methods

### 2.1. Cell Culture, Plasmid, and Transfection

HEK293 (human embryonic kidney cell line, obtained from the Weihong Song Library, from University of British Columbia, Vancouver, BC, Canada) cells were cultured in glucose Dulbecco’s Modified Eagle Medium (DMEM, Thermo Fisher, Beijing, China) containing 10% fetal bovine serum (FBS, Gibco, Grand Island, NY, USA). SH-SY5Y (human neuroblastoma cell line, obtained from the Weihong Song Library) cells were cultured in DMEM/F12 (Thermo Fisher, Waltham, MA USA) with 10% FBS. All cells were maintained at 37 °C in an incubator containing 5% CO_2_. pCDNA4-Rab21-myc/his plasmid was constructed by PCR using cDNA from Hela cells. A ubiquitin plasmid pHis-Ubi without myc tag was derived from pCW-7 [21]. For transient transfection, HEK293 cells were grown to approximately 70% confluence in a 6-well plate and transfected with an indicated plasmid (1 μg each) using Lipofectamine 2000 (Invitrogen, Carlsbad, CA, USA) according to the manufacturer’s instructions. At indicated time of post-transfection, the media was replaced with drugs-containing DMEM. The cells were harvested 48 h after transfection for the following Western blot analysis

### 2.2. Immunofluorescence

Cells cultured in 20 mm glass-bottom confocal dishes (Corning, New York, NY, USA) were fixed with 2% paraformaldehyde at room temperature for 20 min, followed by permeabilization with pre-warmed 0.2% Triton X-100 in PBS at room temperature for 5 min. The cells were then blocked with blocking solution (2% FBS, 2% BSA in PBS) for 30 min before primary antibody incubation. Hoechst 33258 (Beyotime, Shanghai, China) was used to detect the nucleus. Cells were then incubated with rabbit anti-ubiquitin primary antibody (1:1000, Abclonal, Wuhan, China), mouse anti-beclin1 (1:200, Proteintech, Wuhan, China), rabbit anti-LC3 (1:200, Proteintech, Wuhan, China), rabbit anti-Rab21 (1:200, Abclonal, Wuhan, China), and mouse anti-Rab21 (1:100, Santa Cruz Biotechnology, Santa Cruz, CA, USA) in blocking solution for 4 h at room temperature. Alexa FluoroR 488 or 555 secondary antibody (as indicated, Abcam, Cambridge, UK) for 2 h and Hoechst 33258 were used for nucleic staining. Cells were imaged with a Nikon A1R SI confocal microscope (Nikon, Tokyo, Japan).

### 2.3. Western Blotting

Cells were sonicated with a sonicator (Sonics, Newtown, CT, USA) (10 pulses on ice, 20% amplitude) and centrifuged (14,000× *g*, 20 min) in the lysis buffer. Protein samples obtained from cell lysate were mixed with 4× loading buffer, boiled at 100 °C for 5 min, and subjected to SDS-PAGE. Protein samples were separated in 15% Tris-glycine gels and transferred to a PVDF membrane (Millipore, Billerica, MA, USA). After being blocked with 5% skimmed milk in TBS Tween-20 (TBST, 0.1% Tween-20, Solarbio, Beijing, China), membranes were incubated with the indicated primary antibodies diluted in TBST at 4 °C overnight later incubated with HRP-conjugated secondary antibody at RT for 2 h. The image was detected using the Bio-Rad ChemiDoc XRS^+^ system (Bio-Rad, Hercules, CA, USA).

### 2.4. Statistical Analysis

All values are presented as mean ± S.E.M. Data analysis was conducted by GraphPad Prism 6 (GraphPad Software, La Jolla, CA, USA). Student’s *t*-test was used to calculate the *p*-value of each independent experiment between Rab21 and the treated group. *p*-value < 0.05 was regarded significant. Regression fitting by ELISACalc.exe (Blue Gene, Shanghai, China).

## 3. Results

### 3.1. Rab21 Is Degraded by Proteasome- and Lysosome-Dependent Proteolysis

**Figure 1:**
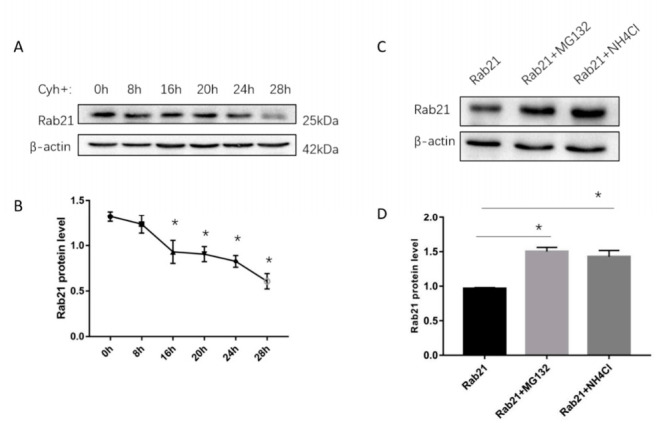
The time-pulse chase assay for the half-life and the proteasome/lysosome-mediated degradation of Rab21 protein. (**A**) Rab21 plasmid (pcDNA4-Rab21) was transfected into HEK293 cells using Lipofectamine 2000. The transfected cells were treated with 100 μmol/L cycloheximide for 0, 8, 16, 20, 24, 28 h and then harvested. Rab21 proteins were detected by Western blot. (**B**) The protein level of Rab21 was shown by a line chart. (**C**) Rab21 plasmid transfected HEK293 cells were treated with vehicle solution control or 10 μmol/L MG132 or 10 mmol/L ammonium chloride for 24 h. (**D**) Rab21 protein levels were significantly increased by proteasomal and lysosomal inhibitor treatment. Protein levels were normalized to β-actin controls. Data are presented as mean ± SD. *N* = 3. * *p* < 0.05 relative to control by ANOVA.

To find out how Rab21 is degraded, we initially examined the half-life of endogenous Rab21 by using the cycloheximide (Cyh) chasing assay. The HEK293 cells were treated with 100 μmol/L Cyh and collected at different time points. The protein level of Rab21 was gradually decreased along with the increased time of Cyh addition, which was reduced to roughly a half at 27.24 h by regression fitting calculation (Figure 1A,B). Then, we examined whether the degradation of Rab21 protein is mediated by the proteasome- or lysosome-mediated pathway. The HEK293 cells were transfected with Rab21 plasmid (pcDNA4-Rab21) and were further treated with proteasome or lysosome inhibitors. The Rab21 protein level was examined by Western blotting. With the addition of the proteasome inhibitor MG132, the protein level of Rab21 was significantly increased by 48%. Similarly, treatment of the lysosome inhibitor ammonium chloride (NH_4_Cl) also induced Rab21 protein to a similar level (46%) when compared to the control (Figure 1C,D). These results suggest that the degradation of Rab21 might be controlled by proteasome and lysosome.

**Figure 2:**
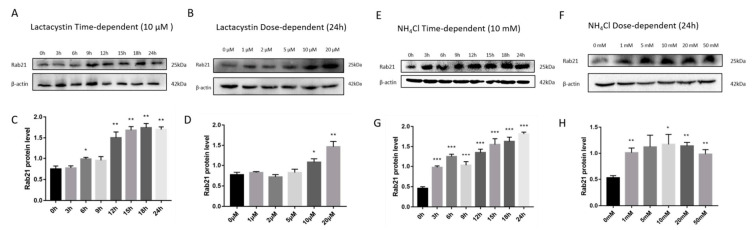
The proteasome- and lysosome-dependent proteolysis of exogenous Rab21. (**A**) Rab21 plasmid (pcDNA4-Rab21) was transfected into HEK293 cells using Lipofectamine 2000. The transfected cells were treated with 10 μmol/L lactacystin for 0–24 h for a time-course assay. (**B**) Rab21 plasmid transfected HEK293 cells were treated with vehicle or lactacystin with gradient concentrations (0, 1, 2, 5, 10, 20 μmol/L) for 24 h. Rab21 protein levels were gradually increased in a lactacystin time- (**C**) and dosage- (**D**) dependent manner. (**E**) Rab21-containing plasmid transfected HEK293 cells were treated with 10 mmol/L ammonium chloride for 0–24 h’s time-dependent assay. (**F**) Rab21 plasmid transfected Hek293 cells were treated with gradient concentration (0, 1, 5, 10, 20, 50 mmol/L) of ammonium chloride for 24 h. Rab21 protein levels gradually increased in ammonium chloride in a time- (**G**) and dose- (**H**) dependent manner. Data are presented as mean ± SD. *N* = 3. * *p* < 0.05, ** *p* < 0.01, *** *p* < 0.001 relative to control by ANOVA.

To further determine the degradation pathway of Rab21 protein, we also applied lactacystin, a highly specific and irreversible proteasome inhibitor, by specifically targeting the 20S proteasome while not interfering with lysosome-related degradation. We transfected HEK293 cells with a plasmid containing Rab21 for 48 h and then treated cells with different concentrations of lactacystin. Lactacystin treatment significantly increased Rab21 protein levels in a dose-dependent and time-dependent manner (Figure 2A–D). NH_4_Cl is a weak base known to disrupt lysosomal function by alkalizing the intralysosomal pH value. Similarly, HEK293 cells containing Rab21 plasmid were treated with gradient concentrations of NH_4_Cl after 48 h cell transfection. Consistently, the protein level of Rab21 was dramatically induced both dose-dependently and time-dependently (Figure 2E–H). These results further confirm that the degradation of Rab21 was modulated by both the proteasome- and the lysosome-mediated pathway.

**Figure 3:**
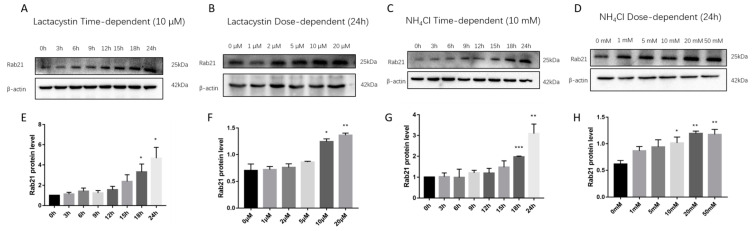
The proteasome- and lysosome-dependent proteolysis of endogenous Rab21 protein (**A**) SH-SY5Y cells were treated with 10 μmol/L lactacystin for 0–24 h time-course assay. (**B**) SH-SY5Y cells were treated with vehicle or lactacystin with gradient concentrations (0, 1, 2, 5, 10, 20 μmol/L) for 24 h. Rab21 protein levels in the lactacystin time- (**C**) and dosage- (**D**) dependent experiments were significantly increased. (**E**) Cells were treated with 10 mmol/L ammonium chloride for 0–24 h time-dependent assay. (**F**) Cells were treated with gradient concentrations (0, 1, 5, 10, 20, 50 mmol/L) of ammonium chloride for 24 h. Rab21 protein levels in the ammonium chloride time- (**G**) and dosage- (**H**) dependent experiments were significantly increased. Data are presented as mean ± SD. *N* = 3. * *p* < 0.05, ** *p* < 0.01, *** *p* < 0.001 relative to control by ANOVA.

To testify whether endogenous Rab21 was also degraded by proteasome or lysosome, we treated SH-SY5Y cells with lactacystin and NH_4_Cl. Consistent with the results of exogenous Rab21 protein degradation, the endogenous Rab21 protein level was remarkably increased with treatment of lactacystin from 18 to 24 h with graded concentrations of 10 and 20 µM. NH_4_Cl treatment showed similar changes of endogenous Rab21 protein, which is also in a dose-dependent and time-dependent manner (Figure 3A–H). These data suggest Rab21 is endogenously degraded by proteasome- and lysosome-dependent proteolysis.

### 3.2. Rab21 Is Ubiquitinated

**Figure 4:**
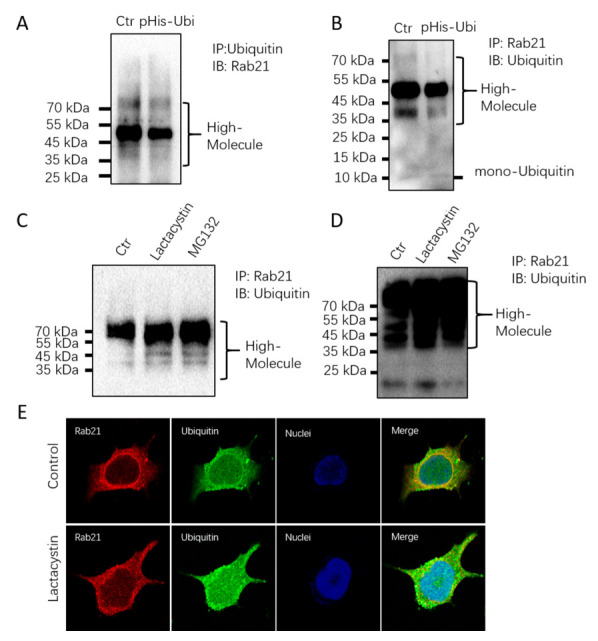
The proteasome-mediated degradation of Rab21 is ubiquitin-dependent. (**A**) Hek293 cells were transfected with Rab21 plasmid or pCDNA4 plasmid for 48 h. Cell lysates were immunoprecipitated with anti-ubiquitin followed by immunoblotting with anti-Rab21. (**B**) Hek293 cells were transfected with Rab21 plasmid or pCDNA4 plasmid for 48 h. Cell lysates were immunoprecipitated with anti-Rab21 followed by immunoblotting with anti-ubiquitin. (**C**,**D**) SH-SY5Y cells were treated with either vehicle or lactacystin or MG132 for 24 h. Cell lysates were immunoprecipitated with anti-Rab21 followed by immunoblotting with anti-ubiquitin. (**E**) SH-SY5Y cells were treated with either vehicle or lactacystin (10 μmol/L) for 24 h. Cells were fixed and incubated with primary antibody of Rab21 and ubiquitin, then incubated with Alexa Fluor^®^ 488 (Abcam, Cambridge, UK) conjugated donkey anti-rabbit secondary antibody and Alexa Fluor^®^ 555 (Abcam, Cambridge, UK) conjugated donkey anti-mouse secondary antibody. Proteasomal inhibitor treatment caused accumulation of ubiquitinated Rab21. Scale bar = 10 μm. Magnification: ×100.

Ubiquitination is a hallmark for proteins that are degraded by proteasome-dependent proteolysis. The above data has approved that proteasome inhibition leads to increased Rab21 protein level both exogenously and endogenously, which raised the question of whether Rab21 is ubiquitinated. To do so, we transfected HEK 293 cells with a plasmid containing ubiquitin. The co-immunoprecipitation (CO-IP) assay displayed that Rab21 can be detected by Rab21 antibody when ubiquitin was used for immunoprecipitation; similarly, when Rab21 was used for immunoprecipitation, Rab21 can also be detected by ubiquitin antibody, suggesting Rab21 can interact with ubiquitin (Figure 4A,B). When cells are treated with lactacystin and MG132, the CO-IP data showed that the ubiquitination of Rab21 was much increased (Figure 4C), suggesting the proteasome degradation of Rab21 is ubiquitin-dependent. The immunofluorescence assay also showed ubiquitin can be co-localized with Rab21 in SH-SY5Y cells, while with lactacystin treatment, the colocalization between Rab21 and ubiquitin was remarkably increased (Figure 4D,E). These further confirmed the ubiquitination-dependent proteasome degradation of Rab21.

### 3.3. The Ubiquitination of Rab21 Is Increased in Alzheimer’s Disease (AD) Model Mice

**Figure 5:**
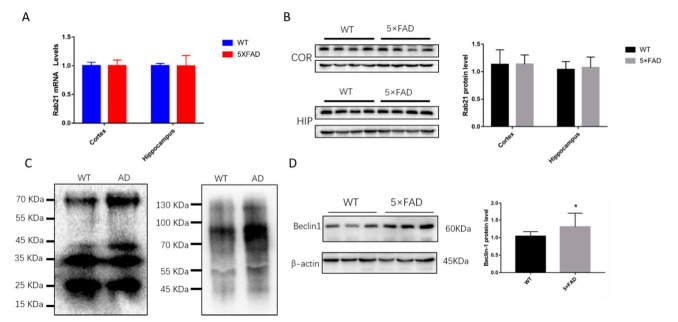
The level of Rab21 and the ubiquitination of Rab21 in AD model mice (**A**) Rab21 mRNA levels in the brain of WT and 5 × FAD mice were detected by real-time PCR. Rab21 mRNA quantifications were normalized to β-actin. (**B**) Rab21 protein levels in the cerebral cortex and hippocampus of WT and 5 × FAD mice were examined by Western blot. (**C**) Lysates of the cerebral cortex were immunoprecipitated with anti-ubiquitin followed by immunoblotting with anti-Rab21. (**D**) Beclin1 protein level in the hippocampus of WT and 5 × FAD mice were examined by Western blot. Data presented as mean ± SD. *N* = 4 for WT and *N* = 4 for 5 × FAD mice (9 months old). Statistical analyses were performed using Student’s *t*-test. * *p* < 0.05 relative to control by ANOVA.

Our previous study also indicated that Rab21, functioning as a PS1 interactor, cargos PS1 from early endosomes to late endosome/lysosome to perform its role for APP hydrolysis. Whether the level of rab21 or the ubiquitination of Rab21 is changed in AD model mice is still unknown. Compared to WT mice, the mRNA level or the protein level of Rab21 was kept unchanged in the cortex or hippocampus of the 5 × FAD mice (Figure 5A,B), suggesting the Rab21 itself was not affected in AD. Many studies have reported that the proteasome degradation system was impaired in AD. Interestingly, we found that the ubiquitination of Rab21 in AD was much increased compared to that in WT mice (Figure 5C). Furthermore, the protein level of Beclin1, the biomarker of the autophagy-lysosome pathway, was found to be increased in AD model mice (Figure 5D), indicating the autophagy-lysosome pathway may function in parallel to degrade Rab21 protein.

### 3.4. Rab21 Upregulate Autophagy-Related Genes

**Figure 6:**
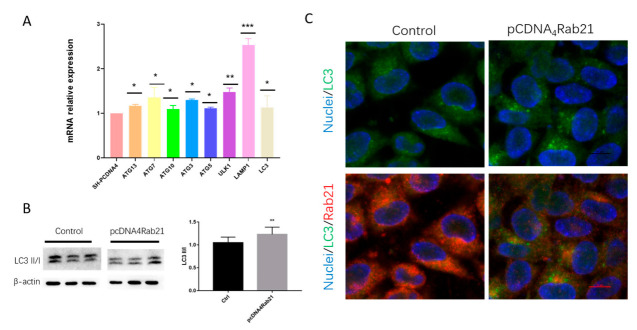
Rab21 upregulates genes involved in the autophagy-lysosome pathway. (**A**) SH-SY5Y cells were transfected with Rab21 plasmid for 48 h. Autophagy-lysosome pathway-related genes were detected by real-time PCR. (**B**) Cell lysates were immunoblotted with anti-LC3. LC3 II/I was significantly increased by overexpression of Rab21. (**C**) SH-SY5Y cells were transfected with Rab21 plasmid for 48 h. Cells were fixed and incubated with primary antibody of Rab21 and LC3, then incubated with Alexa Fluor^®^ 488 conjugated donkey anti-rabbit secondary antibody and Alexa Fluor^®^ 555 conjugated donkey anti-mouse secondary antibody. Scale bar = 10 μm. Magnification: ×100. Data are presented as mean ± SD. *N* = 3. * *p <* 0.05, ** *p <* 0.01, *** *p <* 0.001 relative to control by ANOVA.

The above data showed that the ubiquitinated Rab21 increased in 5 × FAD mice, but the total protein level remained unchanged. It is known that the proteasome function was severely impaired in AD. We speculated that the autophagy-lysosome pathway works in parallel to degrade Rab21, thus maintaining its protein quality control. Autophagy is a highly conserved biological process in all eukaryotes that target macromolecules for lysosome-mediated degradation to control cell homeostasis [22]. Therefore, we overexpressed Rab21 in SH-SY5Y cells and found that a series of genes involved in the autophagy pathway, including ULK1, Atg7, Atg10, Atg13, Atg5, Beclin1, Lamp1, and LC3, all showed increased levels under overexpression of Rab21 (Figure 6A). In particular, we further examined the protein level of LC3II/I and found that LC3II/I was increased by overexpression of Rab21 (Figure 6B). The immunostaining result also showed that the green fluorescent spots are increased dramatically in the Rab21 group compared to the control, suggesting the production of LC3II is increased by overexpression of Rab21 (Figure 6C). These data suggest Rab21 can trigger the activation of the autophagy-lysosome pathway that may play a key role in keeping its homeostasis.

## 4. Conclusions and Discussion

Alzheimer’s disease (AD) is an age-related neurodegenerative disorder, with main clinical symptoms of progressive memory impairment, cognitive impairment, a decline in daily living ability, and mental disorders. The Aβ, produced through sequential cleavage of APP by β- and γ-secretases, has been considered as one of the main hallmarks of the neuropathogenesis of AD. Many Rabs are reported to be associated with AD. Rab11 is identified as a key regulator for controlling β-secretase endosomal recycling to the plasma membrane and thus affecting Aβ production [23]. Silencing *RAB44*, *RAB6A,* or *RAB10* results in a decreased level of Aβ without affecting the activity of β-secretase [23]. Rab1A and Rab7A are functionally linked to tau secretion, which is also an important pathological marker of AD [24,25]. Our previous work has demonstrated that Rab21 regulates the activity of gamma-secretase via interacting with PS1 (the main catalytic subunit of γ-secretase) instead of affecting the expression of gamma-secretase, suggesting that Rab21 protein may be a potential target for AD treatment [20]. In this study, we focused on the Rab21 protein itself and revealed the proteolytic pathways of Rab21. Interestingly, not only degraded by a ubiquitin-proteasome pathway, but Rab21 can also be degraded by the autophagy-lysosome pathway. In particular, under the condition of AD, when the ubiquitin-proteasome system is impaired, the autophagy-lysosome pathway could play a role in supplementing the function of the ubiquitin-proteasome system to degrade Rab21 and ensure the homeostasis of Rab21 protein in the brain.

The ubiquitin-proteasome pathway and autophagy-lysosome pathway are two predominant intracellular mammalian degradation pathways that modulate protein quality control and maintain cellular homeostasis [26]. The ubiquitin-proteasome pathway regulates a series of critical cellular functions, including differentiation, proliferation, and apoptosis. Changes in this pathway may lead to developmental abnormalities, autoimmune diseases, neurodegenerative diseases, and cancer [27,28]. Similarly, the autophagy-lysosome pathway can be significantly upregulated by many cell stressors and diseases [29]. The ubiquitin-proteasome pathway is primarily responsible for degrading short-lived, soluble unfolded, and misfolded proteins.

In contrast, the autophagy-lysosome pathway is mainly recruited for digesting a variety of bulky substrates, including long-lived proteins, protein complexes, oligomers, insoluble aggregates, and even abnormal organelles [30]. These two proteolytic pathways are distinct in mechanism but are inextricably connected. It is reported that when the ubiquitin-proteasome pathway is inhibited or overburdened, the autophagy-lysosome pathway will be stimulated for sequestering cellular materials into autophagosomes and trafficking to lysosomes for degradation [29]. Furthermore, many proteins can be degraded by the ubiquitin-proteasome pathway and the autophagy-lysosome pathway, forming a single network for sustaining protein equilibrium [26,29].

In our study, we found Rab21 protein can be degraded by both the ubiquitin-proteasome pathway and the autophagy-lysosome pathway. In particular, ubiquitinated-Rab21 is upregulated while the total protein level of Rab21 is unchanged in the hippocampus of AD model mice, suggesting ubiquitinated-Rab21 cannot be degraded by an impaired proteasome system but be degraded by the autophagy-lysosome pathway, thus keeping its quality control in a balanced condition. This is further confirmed by the result that overexpression of Rab21 leads to the upregulation of a series of genes involved in the autophagy-lysosome pathway.

The autophagy-lysosome pathway can be divided into several stages, including activation of autophagy initiation complexes, nucleation of phagophores, phagophore extension, autophagosome maturation, autophagosome-lysosome fusion, and lysosomal-mediated degradation [31]. Multiple protein complexes are required to manipulate autophagosome trafficking along microtubules and connect autophagosomes to lysosomes [29]. Autophagy initiation is modulated by two ser/thr kinases, the ULK1 (UNC-51-like kinase 1) and mTOR (mammalian target of rapamycin) [29]. ULK1 and ATG13, ATG101 and FIP200 (family kinase interacting protein of 200 kDa) form the initiation complex for autophagy initiation [32]. Beclin1 can be phosphorylated by ULK1, which can recruit genes to form the PI3K complex responsible for nucleating phagophore assembly in the nucleation stage [33,34]. ATG12, ATG10, and ATG5 form a ubiquitin-like conjugation system are involved in the elongation stage. In addition, the post-translation modification of LC3 protein occurs in the elongation stage that produces LC3I and LC3II. The elongation is controlled by the ATG12-ATG5-ATG16L1 complex inserting LC3II into phagophores. In addition, LC3II level is changed in autophagosome maturation; thus, the ratio of LC3II/I has been considered a key value for estimating the autophagy-lysosome pathway [35,36,37]. Our work displayed that the associated genes involved in the autophagy-lysosome pathway, such as ULK1, Lamp1, ATGs, and LC3, are induced significantly by overexpression of Rab21, suggesting overexpressing Rab21 can trigger the activation of the autophagy-lysosome pathway that may act as a complementary mechanism to keep Rab21 in homeostasis.

In conclusion, our work firstly revealed that both the ubiquitin-proteasome pathway and the autophagy-lysosome pathway are involved in the degradation of Rab21 protein. It needs to be highlighted that these two proteolytic pathways function together to maintain the protein quality control of Rab21. In the AD model, ubiquitinated Rab21 could not be fully degraded by the proteasome pathway; however, the autophagy-lysosome pathway works complementary to degrade Rab21 and maintain its homeostasis. However, it is still unclear how ubiquitinated Rab21 would be degraded by the autophagy-lysosome pathway, or which regulator would cargo ubiquitinated Rab21 to the lysosome could be further studied. Considering the vital role of Rab21 in membrane trafficking and its involvement in diseases, understanding the characters of Rab21 protein itself will help explore its potential of being a target for disease therapeutic strategy.

## Data Availability

The data presented in this study are available on request from the corresponding author.
